# Mobility and Balance and Their Correlation with Physiological Factors in Elderly with Different Foot Postures

**DOI:** 10.1155/2015/385269

**Published:** 2015-10-25

**Authors:** Aisyah Mohd Said, Haidzir Manaf, Saiful Adli Bukry, Maria Justine

**Affiliations:** Department of Physiotherapy, Faculty of Health Sciences, Universiti Teknologi MARA, Puncak Alam Campus, 42300 Puncak Alam, Selangor, Malaysia

## Abstract

This study determines (1) the correlation between mobility and balance performances with physiological factors and (2) the relationship between foot postures with anthropometric characteristics and lower limb characteristics among elderly with neutral, pronated, and supinated foot. A cross-sectional observational study was conducted in community-dwelling elderly (age: 69.86 ± 5.62 years). Participants were grouped into neutral (*n* = 16), pronated (*n* = 14), and supinated (*n* = 14) foot based on the foot posture index classification. Anthropometric data (height, weight, and BMI), lower limb strength (5-STS) and endurance (30 s chair rise test), mobility (TUG), and balance (FSST) were determined. Data were analyzed using Spearman's correlation coefficient. Body weight was negatively and moderately correlated (*r*_*s*_ = −0.552, *P* < 0.05) with mobility in supinated foot; moderate-to-high positive linear rank correlation was found between lower limb strength and mobility (*r*_*s*_ = 0.551 to 0.804, *P* < 0.05) for pronated and neutral foot. Lower limb endurance was negatively and linearly correlated with mobility in pronated (*r*_*s*_ = −0.699) and neutral (*r*_*s*_ = −0.573) foot. No correlation was observed in balance performance with physiological factors in any of the foot postures. We can conclude that muscle function may be the most important feature to make movement possible in older persons regardless of the type of foot postures.

## 1. Introduction

The foot is an important body part because it supports body weight and organizes locomotion. However, this body part is vulnerable to daily strains when an individual walks [1]. Musculoskeletal disorders, such as foot malalignment, may be associated with functional restraint, even though a particular disorder is not painful [2]. Foot characteristics are related to mobility and functionality in elderly [3]. The present study focuses on the dynamic foot function and gait performances concerning individuals of advanced age rather than the type of foot. In theory, the structure of the lower limb and foot may be vulnerable to several factors, such as footwear [4–6], excessive body weight [7], job nature [8], and physical activity level [9]; these factors may affect foot structures as people age. These factors may also influence some lower limb functions, such as balance and gait performances.

Older persons with foot problems have reported multiple falls compared with those who do not have foot problems; this phenomenon may indicate a higher risk of fall in the future [10]. For instance, individuals with pronated feet are at a high risk of falls or loss of balance during unilateral stance in functional activities; individuals with supinated feet may present disturbed postural control [11]. The lower limb function is also affected by foot posture; for example, in contrast to individuals with normal-arched feet, individuals with flat-arched feet use their tibialis anterior muscle during the contact phase but use the tibialis posterior muscle during midstance or propulsion [12]. Differences in muscle activity may be a sign of a neuromuscular compensation to decrease the overwork of the medial longitudinal arch. These differences may lead to other problems, such as muscular fatigue, which eventually affects balance performance during dynamic activities [13–15].

This study aimed to address the problems regarding foot posture and balance performances in elderly. Some variations in foot posture are associated with changes in lower limb motion and muscle activity, which are strongly influenced by several systemic conditions, such as neurological or rheumatological diseases [16]. Foot posture also affects the mechanical alignment and dynamic function of the lower limb; therefore, foot posture may be related to the development of lower limb disorders [17]. Furthermore, our research scope is similar to those described in previous studies [11, 18–20]. Significant changes have been observed in functional performances among different foot postures; however, the tested population is limited to young adults. With ongoing interests, whether different types of foot postures affect mobility and balance of the elderly remains inconclusive; elderly possibly exhibit high severity partly because of the aging process.

This study aimed to (1) determine whether physiological factors, such as anthropometric data (height, body weight, and body mass index (BMI)) and lower limb characteristics (strength and endurance), are associated with balance and mobility in elderly with different types of foot postures, that is, neutral, pronated, and supinated feet; this study was also conducted to (2) determine the relationship of foot postures with anthropometric characteristics and lower limb characteristics. The result of this study may provide the basis of extensive studies on the assessments of foot posture in clinical settings. This study may also be applied to identify lower limb conditions in the early stages and predict the risk of falls. We hypothesized that the anthropometric data and lower limb characteristics were significantly correlated with balance and mobility regardless of the types of foot postures. We also hypothesized that foot posture may significantly differ in anthropometric and lower limb characteristics.

## 2. Methods

### 2.1. Participants and Study Design

This study applied a cross-sectional design. Power analysis [11, 18–20] was performed to compare foot postures and functional performances of individuals with different foot types; in this method, an estimated sample of 30 to 48 participants could provide significant results. Thus, 44 community-dwelling elderly females (age range = 60 years to 85 years) were recruited via a convenience sampling to yield a significant result. The participants were included if the following criteria were satisfied: (1) there are no chronic orthopedic conditions, such as rheumatoid arthritis, severe knee osteoarthritis, and acute fracture, injury, or pain in the lower limb area; (2) there are no vestibular or neurological impairments; (3) there is no peripheral neuropathy or sensory deficits caused by diabetes or any systemic conditions; (4) they can walk continuously for 10 m without walking aids; and (5) they are not involved in any structured exercise classes of more than three times a week (physically inactive). All participants included in the study signed an informed consent approved by the institutional ethics committee. All three subgroups (pronated, supinated, and neutral feet) were formed from the total eligible participants. On the basis of the assessment, we found that 16 patients (age 65 years to 81 years) exhibited neutral feet, 14 patients (age 60 years to 80 years) presented pronated feet, and 14 patients (age 61 years to 85 years) manifested supinated feet. All the participants were actively involved in religious classes 3 to 5 times per day, every day, which were held at the mosque located at least 20 to 500 metres from their home.

### 2.2. Study Procedures and Outcome Measures

The test procedure was performed indoors, in a controlled environment. The feet of the participants were examined by one assessor using the six-item foot posture index (FPI), a clinical diagnostic tool that can distinctively quantify and classify the particular foot as neutral, pronated, or supinated posture [21]. The FPI reliability coefficient of the application on elderly is 0.61 [22].

#### 2.2.1. Anthropometric Data

Anthropometric factors, including height (m), weight (kg), and BMI (kg/m^2^), were evaluated, in accordance with a standard procedure.

#### 2.2.2. Lower Limb Characteristics (Lower Limb Strength and Endurance)

The five-time sit-to-stand test (5-STS) was used to measure lower limb strength [23]. The participants were initially instructed to be in a sitting position on a chair with a standard height of 45 cm from the ground [24]; in a sitting position, the participants were also instructed to have both of their arms crossed at the wrists and placed on the chest. The test required the participants to stand and sit repeatedly five times as fast as possible; during this test, the researcher used a stopwatch and recorded the time (s) at which the task was completed. A short time to complete the test corresponds to a strong lower limb.

Lower limb endurance was measured using the 30 s chair rise test [25]. In this test, the participants were instructed to stand upright from a chair and to sit again with their arms folded across their chest. This task was performed repeatedly or as much as they can in their self-preferred speed for 30 s. Numerous repetitions from a sitting position to a standing position indicate excellent lower limb endurance.

#### 2.2.3. Mobility

The Timed-Up and Go test (TUG) was used to measure the mobility of the participants. Several studies [1, 4, 26, 27] have applied this test, particularly in elderly populations and patients with neurological conditions. TUG has also demonstrated good interrater reliability (ICC = 0.99) [28] in elderly when this parameter is used to assess functional mobility. The testing procedure [29] required the participant to stand from a seated position on a standard chair with a seat height of approximately 40 cm to 50 cm, walk at a normal walking speed along a 3 m distance, and turn and walk back toward the chair to sit again. TUG was chosen to reflect mobility based on its characteristic that involves “transition” of multiple activities of sit-to-stand, walking at short distances, and changing direction [30]. A short time to complete the test indicates good functional mobility.

#### 2.2.4. Balance

The Four-Square Step test (FSST) was used to measure balance performance. This test can be used as a reliable and valid tool to assess the dynamic standing balance of older people, including those with transtibial amputation or those with vestibular dysfunctions [31]. The stepping base was constructed using two canes crossed with each other; four squares numbered from 1 to 4 were formed on the floor. The participants performed this test in the following stepping sequence: clockwise starting at square 1, proceeding to squares 2, 3, and 4, and back to square 1; counterclockwise starting at square 4, proceeding to squares 3 and 2, and ending at square 1. Both feet must be placed in each square as the participants moved from one square to another. The score was recorded as the time spent to complete the sequence. The stopwatch started when the first foot contacted the floor in square 2 and ended when the last foot came back to touch the floor in square 1 [32]. The test was repeated if the participant failed to complete the sequence, lost balance, or came in contact with the cane. A good balance performance is indicated by a short time in seconds to complete the task.

### 2.3. Statistical Analysis

Descriptive statistics and correlation analysis were performed using SPSS 20.0 (IBM Corporation, Somers, NY). The mean and standard deviation were calculated for each variable. The significance level was set as a priori at *P* < 0.05. Comparisons between foot posture (neutral, pronated, and supinated) with anthropometric characteristics and lower limb characteristics were tested using Kruskal-Wallis analysis (nonparametric). Neutral, pronated, and supinated feet as subgroups were analyzed using Spearman's correlation coefficients to determine the associations of the physiological domains with balance and mobility. All analyses were done using nonparametric test. The correlation results were interpreted as poor (*r*_*s*_ < 0.30), low (*r*_*s*_ = 0.30 to 0.50), moderate (*r*_*s*_ = 0.50 to 0.70), high (*r*_*s*_ = 0.70 to 0.90), or very high (*r*_*s*_ > 0.90) [33].

## 3. Results

### 3.1. Demographic Data, Physiological Factors, and Mobility and Balance in Different Types of Foot Postures

Table 1 presents the demographic data, physiological factors, and mobility and balance among the participants. The results of mean comparisons revealed that none of the variables was significantly different from one another among all of the groups of elderly in terms of foot postures.

### 3.2. Correlation of Mobility and Balance

Spearman's correlation coefficients (Table 2) indicated that height and BMI were not correlated with balance or mobility in any types of foot. However, weight was moderately and negatively correlated with mobility in the supinated feet (*r*_*s*_ = −0.552, *P* < 0.05); by contrast, weight was not correlated with pronated and neutral foot group. Lower limb strength was significantly and moderately correlated with mobility in elderly with pronated feet (*r*_*s*_ = 0.551); lower limb strength also exhibited a significantly and highly positive correlation with mobility in elderly with neutral feet (*r*_*s*_ = 0.804, *P* < 0.01). Furthermore, lower limb endurance was highly and negatively correlated with mobility in the pronated feet (*r*_*s*_ = −0.669, *P* < 0.01), while lower limb endurance was negatively and moderately correlated with mobility in the neutral feet (*r*_*s*_ = −0.573, *P* < 0.05). Balance performance was not correlated with any of the anthropometric or physiological factors in all of the three types of foot.

## 4. Discussion

This study aimed to determine the associations of balance and mobility with physiological factors in elderly with neutral, pronated, and supinated foot postures. To our knowledge, studies have extensively investigated the relationship of physiological factors with balance and mobility; however, these studies have not implemented the commonly used senior fitness test to represent the physiological characteristics of participants [34, 35]. We believed that the implementation of the measurement tools in this study could represent the basic movement that is more functional for elderly rather than the use of advanced technologies. This study is the first to demonstrate the direct relationship of balance variables with physiological and anthropometric factors among elderly with different types of foot postures.

### 4.1. Anthropometric, Mobility, and Balance

In this study, height was not correlated with mobility in all types of foot. This result is not consistent with that described in a previous study [36], which revealed that height is correlated with balance variables, such as static standing; increased height corresponds to poor balance. This finding is attributed to the center of mass [36, 37] and the increase in the response of ankle and gastrocnemius as height increases [38]. Thus, muscle activation may explain the findings of the current study. In neutral and supinated foot groups, the intrinsic factors that influenced foot arch might also affect the ankle range of movement and agility, especially during turning in the TUG. However, this theory should be verified through a biomechanical analysis or electromyography (EMG) to accurately determine the involvement of intrinsic musculatures.

Our results also demonstrated that weight was associated with the mobility of individuals with supinated feet; by contrast, weight was not associated with the balance and mobility of individuals with other types of foot. However, this finding is inconsistent with that in previous studies, which demonstrated a decrease in balance in individuals with a heavier body weight compared to those with a lighter body weight [34, 39, 40]. Indeed, body weight is a reliable predictor of mobility and balance [40]. The inconsistency between the present study and a previous study [34] can be due to the differences in the mean age and BMI. In a previous study [39], underweight, normal weight, overweight, and obese individuals are compared in terms of stability; the results show that body weight is correlated with stability. Weight gain induced changes in stability regardless of gender; in particular, individuals in the underweight group demonstrate a good balance performance; postural activity is inversely proportional to increased BMI. These inconsistencies could also be attributed to the different physiological factors of the participants in the studies; for instance, the mean age, BMI, and number of participants in each study differed (Table 3). In our study, our unexpected results could be attributed to the small sample size.

A previous study [39] explained that balance deterioration in the obese group is due to an individual's inability to generate sufficient muscle force to control the displacement mass trajectory during balance performance. However, the results of the previous study [39] demonstrated contrasting findings; in particular, most participants are overweight but characterized with considerable lower limb strength. Individuals who spend more than 13.6 s to rise from a chair for five repetitions likely exhibit increased disability and morbidity [41]. In contrast to previous findings, the present results showed that approximately 74% of the participants completed the 5-STS in less than 13.6 s. The result explains that most of the participants have considerably good lower limb strength regardless of their age and BMI. This result can be attributed to the nature of the physical activities of the participants.

In terms of mobility, which was measured using TUG, the participants in all of the three groups scored an average of 9.85 s to 10.73 s, which was less than the cut-off time of 13 s. For balance performance as tested by the FSST, only the participants with pronated feet scored an average of 16.75 s, with the cut-off time of 15 s [32]. Thus, most of the participants presented good lower limb strength regardless of the body weight and types of feet; therefore, these participants should be able to theoretically generate sufficient force to maintain balance. We related these findings to the nature of the daily activities of the participants. The participants are community-dwellers, independent in their basic activities of daily living, and actively involved in religious activities. The participants also live near a religious center, where they attend daily classes at least three times to five times a day; these participants also walk from their houses to the center daily. These independent folks may be used to walking at such distances. Thus, these participants presented a good function of the lower limb regardless of the types of feet.

### 4.2. Lower Limb Functions, Mobility, and Balance

On the basis of the results of the correlation analysis, we found that a linear rank correlation existed between lower limb strength and balance performances of the pronated and neutral foot groups. Hence, individuals with better lower limb strength may exhibit a higher ability to perform activities in standing or dynamic standing. This result is consistent with that in a previous study [42], which revealed that foot disorders are unlikely associated with functional outcomes. Thus, the result of our study may be best explained by the lifestyles of the participants; this way of life could be the confounding factor of balance and mobility rather than the type of foot, especially in women [1].

The lower limb endurance demonstrated a good linear rank correlation with balance and mobility in the neutral and pronated foot groups. Previous studies [24, 43, 44] showed that functional balance and mobility are directly improved as lower limb endurance increased. However, the current study revealed contradicting findings through comparisons among types of foot. In theory, individuals with supinated feet may be less stable because of the intrinsic muscle tightness and reduced medial longitudinal arch that causes less flexibility and less stability than those with neutral feet [45]. TUG test encompasses the elements of standing from sitting and walking at a distance as well as turning [30], where agility may play a role more than strength to complete the test. Thus, the TUG performance cannot be determined regardless of muscle strength. The pronated and neutral foot groups showed a greater foot contact area and better intrinsic stability than the supinated foot group; thus, the administration of TUG and the relationships with lower limb endurance yielded better results with less confounding factors.

In the population considered for this study, the confounding factor may be caused by the administration and the psychometric properties of the assessment tool. For instance, TUG is known as an excellent outcome measure to identify the risk of fall; however, TUG is unable to identify the existing impairments in static or dynamic balance skills of an individual [41]. The type of foot malalignment has been determined and grouped accordingly to establish the effects on the participants' performances. This process also applies to the results of balance performance. An ability to maintain balance should be associated with various factors, such as coordination, vestibular system, motor response, sensorimotor, and musculoskeletal characteristics of a particular individual. In the administration of the FSST, only the ability of the participants to plan, step, and change directions across obstacles is evaluated [31]; any change in the foot is not considered. Thus, this test might not be sensitive enough to detect any changes in the foot structure during the administration of the test. Furthermore, this type of outcome measure might not be the best to identify the differences in a performance-based perspective. For this reason, the dynamic balance should be evaluated using technical assessments, such as EMG modalities with a sensor plate. Therefore, foot malalignment of the lower limbs does not have a major role in balance and mobility, as measured by TUG and FSST in relation to some physiological characteristics.

### 4.3. Study Limitations

We noted several limitations of this study. First, the participants in this study were community-dwellers who are actively involved in religious classes three times to five times per day; these participants are used to walking independently around their community. This condition may have led to some effect towards their test result. The small sample size also contributed largely to the lack of strength of this finding. Thus, this study could not be used for a higher level of analysis to evaluate the physiological factors as predictors of balance and mobility due to the nonparametric approach in the analysis. With this limitation, the findings may not be generalized to a larger population. Further studies should be conducted using a larger population with a significant disability, including individuals at risk of falls. The researchers of this study also relied solely on the reliability of the FPI based on a previous study and retained only one researcher to evaluate this measure. We wish to extend our study to explore the muscle activity among different types of foot. We believe that further studies, especially those applying EMG, may enhance the objectivity and accuracy of findings to determine the potential differences among these foot postures.

## 5. Conclusion

Muscle properties such as strength and endurance of the lower limb may be the main factors that can affect mobility performance in elderly, regardless of their types of foot postures and thus may be an important feature to make movement possible in older persons. Thus, activities with the element of strength training should be encouraged among older persons; with this method, these individuals can preserve their basic functions for a prolonged period.

## Figures and Tables

**Table 1:** Characteristics of the participants (*N* = 44).

Characteristics	Neutral	Pronated	Supinated	*P* value
	(*n* = 16)	(*n* = 14)	(*n* = 14)	
	Mean (SD)	Mean (SD)	Mean (SD)	
Age (years)	71.13 (4.674)	67.79 (5.780)	70.50 (6.223)	0.196
Height (m)	1.49 (0.065)	1.50 (0.054)	1.52 (0.056)	0.516
Weight (kg)	54.44 (14.289)	60.24 (14.322)	59.91 (11.99)	0.422
Body mass index (BMI) (kg/m^2^)	24.09 (5.238)	26.41 (5.837)	25.94 (4.584)	0.474
Five-time sit-to-stand (sec.)	12.93 (2.608)	11.93 (3.050)	11.67 (2.786)	0.521
30-second chair rise (rep.)	12.63 (2.446)	12.00 (3.762)	13.36 (3.342)	0.230
Timed-Up and Go (sec)	10.73 (2.566)	10.38 (2.166)	9.85 (2.638)	0.484
Four-Square Step test (sec)	14.33 (4.594)	16.75 (6.427)	13.40 (4.232)	0.291

Comparisons were tested using Kruskal-Wallis analysis (nonparametric).

*P* values were set at a significance level of *P* < 0.05.

**Table 2:** Spearman (*ρ*) correlation coefficients of physiological factors with mobility and balance performances in supinated, pronated, and neutral feet.

Correlates	Mobility (TUG)			Balance (FSST)		
	Neutral	Pronated	Supinated	Neutral	Pronated	Supinated
	*r*_*s*_ (*P* value)	*r*_*s*_ (*P* value)	*r*_*s*_ (*P* value)	*r*_*s*_ (*P* value)	*r*_*s*_ (*P* value)	*r*_*s*_ (*P* value)
Height (m)	−0.395	0.378	−0.402	−0.093	−0.013	−0.365
	(0.130)	(0.182)	(0.154)	(0.733)	(0.964)	(0.200)

Weight (kg)	−0.241	−0.024	**−0.552^∗^**	−0.006	0.130	−0.187
	(0.368)	(0.9.34)	**(0.041)**	(0.983)	(0.658)	(0.523)

BMI	−0.144	−0.122	−0.310	−0.109	0.103	−0.020
	(0.594)	(0.679)	(0.281)	(0.688)	(0.725)	(0.946)

LL strength	**0.804^∗∗^**	**0.551^∗^**	0.484	0.368	0.163	0.491
	**(0.041)**	**(0.041)**	(0.079)	(0.161)	(0.578)	(0.075)

LL endurance	**−0.573^∗^**	**−0.669^∗∗^**	−0.242	−0.243	−0.356	−0.266
	**(0.020)**	**(0.009)**	(0.405)	(0.365)	(0.212)	(0.357)

Comparisons were tested using Spearman correlation coefficient analysis (nonparametric).

^∗^Correlation is significant at the 0.05 level (2-tailed).

^∗∗^Correlation is significant at the 0.01 level (2-tailed).

LL: lower limb.

**Table 3:** Comparisons of mean age, BMI, and number of participants in the present study and previous studies.

Author, year	Mean age (years)	Mean BMI (kg/m^2^)	Total participants
Present study	69.08	25.44	50 (44 female; 6 male)
[40]	40.5	35.2	59 (male only)
[39]	22.1	17.4–33.8^∗^	80 (40 female; 40 male)
[34]	22.8	24.6	108 (68 female; 40 male)

^∗^Comparisons were made between groups of underweight, normal weight, overweight, and obese subjects.
